# Regulatory Functions of PurR in *Yersinia pestis*: Orchestrating Diverse Biological Activities

**DOI:** 10.3390/microorganisms11112801

**Published:** 2023-11-17

**Authors:** Liting Xiao, Junyan Jin, Kai Song, Xiuwei Qian, Yarong Wu, Zhulin Sun, Ziyao Xiong, Yanbing Li, Yanting Zhao, Leiming Shen, Yiming Cui, Wenwu Yao, Yujun Cui, Yajun Song

**Affiliations:** 1 School of Basic Medical Sciences, Anhui Medical University, Hefei 230032, China; xlt15576480220@163.com (L.X.); qxw_2020@163.com (X.Q.); 2 State Key Laboratory of Pathogen and Biosecurity, Beijing Institute of Microbiology and Epidemiology, Beijing 100071, China; jinjunyan97@163.com (J.J.); wuyarong525@126.com (Y.W.); sunzhulin0902@126.com (Z.S.); xiaongzy@163.com (Z.X.); liyanbing@csu.edu.cn (Y.L.); zhaoyanting2121@163.com (Y.Z.); 1581182094@126.com (L.S.); m15762561593@163.com (Y.C.); wwyao@cdc.zj.cn (W.Y.)

**Keywords:** *Yersinia pestis*, *purR*, transcriptional regulation, purine biosynthesis

## Abstract

The bacterium *Yersinia pestis* has developed various strategies to sense and respond to the complex stresses encountered during its transmission and pathogenic processes. PurR is a common transcriptional regulator of purine biosynthesis among microorganisms, and it modulates the transcription level of the *pur* operon to suppress the production of hypoxanthine nucleotide (IMP). This study aims to understand the functions and regulatory mechanisms of *purR* in *Y. pestis*. Firstly, we constructed a *purR* knockout mutant of *Y. pestis* strain 201 and compared certain phenotypes of the null mutant (201-Δ*purR*) and the wild-type strain (201-WT). The results show that deleting *purR* has no significant impact on the biofilm formation, growth rate, or viability of *Y. pestis* under different stress conditions (heat and cold shock, high salinity, and hyperosmotic pressure). Although the cytotoxicity of the *purR* knockout mutant on HeLa and 293 cells is reduced, the animal-challenging test found no difference of the virulence in mice between 201-Δ*purR* and 201-WT. Furthermore, RNA-seq and EMSA analyses demonstrate that PurR binds to the promoter regions of at least 15 genes in *Y. pestis* strain 201, primarily involved in purine biosynthesis, along with others not previously observed in other bacteria. Additionally, RNA-seq results suggest the presence of 11 potential operons, including a newly identified co-transcriptional T6SS cluster. Thus, aside from its role as a regulator of purine biosynthesis, *purR* in *Y. pestis* may have additional regulatory functions.

## 1. Introduction

*Yersinia pestis* is the causative agent of the plague, a highly infectious disease that has caused three global pandemics throughout human history [1,2]. This bacterium possesses the ability to adapt to both flea (26 °C) and mammalian (37 °C) body temperatures, as transmission between these hosts is crucial for its natural life cycle [3]. However, *Y. pestis* can undergo physiological changes in anabolism when exposed to different environments. As a relatively young bacterium that diverged from *Yersinia pseudotuberculosis* approximately 7000 years ago [4,5], *Y. pestis* displays a moderate degree of sequence diversity.

Purine plays a vital role in the survival of microorganisms, and most bacteria rely on de novo synthesis for its production. The *purR* gene is widely present in bacterial genomes and functions as a transcriptional repressor, regulating purine biosynthesis by controlling the expression of the *pur* operon [6,7,8]. The deletion of *purR* had been proven to enhance the metabolic flow of the purine pathway and improve the production of riboflavin in *Escherichia coli*, *Bacillus subtilis*, and *Ashbya gossypii* [9,10,11]. However, in certain bacteria, *purR* serves additional roles beyond its involvement in purine regulation. For instance, in *Escherichia coli*, *purR* contributes to the bacterium’s tolerance to organic solvents and enhances its viability when exposed to them [12]. Moreover, mutations in *purR* have been shown to increase the virulence of *Staphylococcus aureus* [13]. Nevertheless, the precise functions of the *purR* gene in *Y. pestis* remains incompletely understood.

To investigate the functions of the *purR* gene in *Y. pestis*, we compared the adaptabilities of *Y. pestis* strain 201 (201-WT) and its *purR* knockout mutant (201-Δ*purR*). We observed that the deletion of *purR* had minimal impact on the environmental adaptability of *Y. pestis* strain 201. To further elucidate the role of *purR* as a transcriptional regulator, we conducted RNA-seq and assessed the expression of the PurR protein. RNA-seq results revealed significant alterations in the transcription levels of numerous genes across the entire genome upon deletion of *purR*, indicating specific associations between certain genes and *purR* in *Y. pestis*. Moreover, Electrophoretic Mobility Shift Assay (EMSA) results demonstrated that PurR could directly bind to the promoter regions of multiple genes within the *Y. pestis* genome, suggesting its direct regulation of their transcription and expression. Collectively, our findings offer valuable insights into the regulatory function of *purR* in governing gene expression in *Y. pestis*.

## 2. Materials and Methods

### 2.1. Bacterial Strains and Culture Conditions

The strains and plasmids used in this study are presented in Table 1, with the primer sequences listed in Supplementary Table S1. *Y. pestis* strain 201 has an identical genome as strain 91,001, which is highly lethal to mice but avirulent to humans [14,15]. Different culture conditions were employed for *Y. pestis* and *E. coli* throughout the experiment. *Y. pestis* was cultivated in LB (Luria-Bertani) medium at a temperature of 26 °C to mimic the temperature of fleas (the vector of *Y. pestis* in the flea transmission process of plague) [16], whereas *E. coli* was cultured in LB medium at 37 °C. Chloramphenicol (34 µg/mL) was added during the cultivation of the complementation strain harboring the pACYC184 plasmid, while kanamycin (50 µg/mL) was necessary for the growth of the strain carrying the pET28a (+) plasmid. All bacterial experiments were performed in a biological safety cabinet.

### 2.2. DNA Extraction and Amplification

Genomic DNA and plasmids were extracted using the QIAamp DNA Mini Kit (Qiagen, Hilden, Germany) and QIAprep Spin Miniprep Kit (Qiagen, Hilden, Germany), respectively, following the manufacturer’s instructions. The target segments were amplified using PCR with 1.1× GoldenStar mix (Green) (Tsingke Biotechnology Co., Ltd., Beijing, China).

### 2.3. Construction of the Mutant and Complementation Strain

The pDS132 was digested by incubating overnight at 37 °C with *Sph*I and *Sac*I enzymes (LMAI Bio, Shanghai, China) together. The upstream and downstream homology arms of *purR* were then ligated with the linearized pDS132 using 2× Seamless Cloning Mix (Biomed, Beijing, China) at 50 °C for 15 min with a molar ratio of vector to DNA of 1:3, and the recombinant vector was introduced into *E. coli* S17λpir to obtain S17-pDS132-*purR*-del for conjugation with *Y. pestis*.

S17-pDS132-*purR*-del and 201-WT strains were cultured in LB medium at either 37 °C or 26 °C until reaching an OD_620_ of 0.8. After centrifugation at 1900× *g* for 5 min, the pellet of S17-pDS132-*purR*-del (1.5 mL) and 201-WT (100 µL) cultures was resuspended and added dropwise onto a filter membrane (0.45 µm) placed on LB plates. The cells on the filter paper were then incubated overnight at 26 °C, and the resulting eluate was spread onto Yersinia Selective Agar Base plates (Oxoid, Basingstoke, UK) supplemented with chloramphenicol (6.8 µg/mL) and incubated at 26 °C. Conjugations were expected to occur under these conditions and were selected on LB plates containing 7% sucrose. The obtained colonies were further analyzed to confirm the expectant conjugant through PCR amplification and sequencing; the correct sequencing results indicate that *purR* has been successfully knocked out, and this strain has been named 201-Δ*purR*.

For complementation of *purR*, the pACYC184 plasmid was digested with *Hind* III and *BamH* I enzymes (LMAI Bio, Shanghai, China), and the *purR* fragment and linearized pACYC184 were then ligated as described previously. The resulting recombinant vector was introduced into competent *E. coli* DH5α cells. The expected recombinant plasmid was identified using PCR sequencing and extracted before being transferred into 201-Δ*purR*. The resulting transformant was confirmed using PCR sequencing and designated as 201-Δ*purR*-Comp.

### 2.4. Growth Rate Determination

The 201-WT and 201-Δ*purR* strains were cultured until they reached an optical density of 1.0 at OD_620_ (*ca*.2 × 10^8^ CFU/mL). Subsequently, the bacterial cultures were inoculated at a ratio of 1:100 in Erlenmeyer flasks containing 60 mL of either fresh LB or a chemically defined TMH liquid medium [20]. The size of Erlenmeyer flasks was 150 mL. All Erlenmeyer flasks were then placed in a precision cell culture shaker (Zhicheng ZWYF-290, Shanghai, China) set at 26 °C with shaking at 200 rpm, and the OD_600_ values of the cultures were measured hourly. Once all strains entered the decline phase, data were collected and plotted for analysis. Each strain underwent three independent biological replications under identical conditions, and the results were expressed as the mean ± standard deviation of the three biological replicated experiments. The experimental procedure at 37 °C was similar to the above, with the exception that the temperature was adjusted accordingly.

### 2.5. Biofilm Formation Analysis

The 201-WT and 201-Δ*purR* strains were cultured until they reached an optical density of 1.0 at OD_620_ (*ca*.2 × 10^8^ CFU/mL) and stored at 4 °C for 16 h. Subsequently, the cultures were diluted by a factor of 10 and transferred into a 24-well cell culture plate, with 1 mL per well. Six independent biological replicates were established for each strain under identical conditions. The plate was then shaken at either 26 °C or 37 °C for 24 h. After removing the bacterial cultures from the wells, OD_620_ was measured. The wells were gently washed twice with deionized water, and the biofilm was fixed at 80 °C for 15 min. Then, 3 mL of 0.1% crystal violet solution (Solarbio, Beijing, China) was added dropwise to each well. The crystal violet solution was discarded after 15 min staining, and the wells were gently washed three times with deionized water. Subsequently, 2.8 mL of ethanol was added to each well and left at room temperature for 3 h. OD_570_ of the solution was determined after a five-fold dilution. The relative amount of biofilm formation was calculated using the formula: 100 × OD_570_/OD_620_.

### 2.6. Survivability under Stressful Environments

The strains 201-WT and 201-Δ*purR* were cultured until they reached an OD_620_ of 1.0. These strains were then exposed to various stressful conditions, including 0.5 M sorbitol for 0.5 h and 1.5 h, 7.5% NaCl for 1.5 h, cold shock for 24 h, or heat shock for 0.5 h. Bacterial numbers were counted before and after the stimulation to determine the survival rate of these two strains under different stressful conditions. Each strain was subjected to three independent biological replicates, and the results were reported as the mean standard deviation of the three experiments.

### 2.7. Real-Time Cell Analysis (RTCA) Assay

HeLa and 293 cells were cultured in Dulbecco’s Modified Eagle’s Medium (DMEM, Solarbio, Beijing, China) supplemented with 10% fetal bovine serum (FBS), at 37 °C and 5% CO_2_. The baseline measurement was taken using 50 µL of DMEM with 10% FBS on the pre-incubated RTCA iCELLigence system (ACEA Biosciences, San Diego, CA, USA), maintained at same conditions. Subsequently, 5 × 10^3^ cells were added to each well of an E-plate and incubated at room time for 30 min. The cells were then transferred to the RTCA iCELLigence system and incubated overnight [21]. The bottom of the cell culture plate compatible with the RTCA system has electrodes to record cell detachment as cell index (CI). Strains were cultivated in LB until they reached an optical density of 1.0 at OD_620_, after removing the supernatant via centrifugation, the bacterial were resuspended in PBS to an optical density of 1.0 at OD620 (*ca*.2 × 10^8^ CFU/mL). Subsequently, the appropriate volume of bacterial suspension was added at a ratio of MOI = 5 or 10 as calculated. Incubation was continued, and the cell index was measured every 15 min and normalized based on the time point at which bacteria were added. Each strain was subjected to three independent biological replicates under identical conditions.

### 2.8. Survival Curves

The 201-WT and 201-Δ*purR* strains were cultured until they reached an optical density of 1.0 at OD_620_ (*ca*.2 × 10^8^ CFU/mL), and the concentration was adjusted to 3 × 10^4^ CFU/mL with PBS. Female BALB/c mice, aged 8–10 weeks, were randomly divided into three groups (*n* = 10) and intraperitoneally challenged with a 100 µL diluted culture. The control group received an equal volume of PBS via the same injection route. Mouse mortality was monitored daily, and the survival curve was plotted.

### 2.9. RNA-Seq and Quantitative Reverse Transcription PCR (qRT-PCR)

201-WT and 201-Δ*purR* were cultured in LB medium at either 26 °C or 37 °C until reaching an OD_620_ of 1.0, and each strain had three biological replicates. Total RNA was extracted from the bacteria using the PureLink™ RNA Mini Kit (Tiangen, Beijing, China) following the manufacturer’s instructions. After measuring the concentration of total RNA, they were sent to Beijing macro & micro- test Bio-Tech Co., Ltd. (Beijing, China) for sequencing. The company created a cDNA library and used Illumina NovaSeq 6000 for sequencing. The extracted RNA was used to create a cDNA library with at least 2 G raw data. The raw data were trimmed with Trimmomatric software fastp version 0.23.4 to filter adapters and low-quality reads (<Q20).

The genes for qRT-PCR were selected based on the results of the RNA-seq analysis under 26 °C culture conditions, and they were YP_RS00205, *purK*, *purE*, *purT*, *purF*, *cvpA*, *purL*, YP_RS13225, *purM*, *purN*, *purH*, YP_RS20395, *ybtE*, *ybtT*, *ybtU*, *irp1*, and YP_RS10830, respectively. The RNA samples used for RNA-seq were reverse transcribed into cDNA using SynScript III RT SuperMix (Tsingke Biotechnology Co., Ltd., Beijing, China). Linear regression analysis was employed to determine the correlations between the RNA-seq data and the outcomes of qRT-PCR

### 2.10. Expression and Purification of PurR

The pET28a (+) plasmid which contains T7 promoter and the target *purR* fragment were digested with *Hind* III and *BamH* I enzymes (LMAI Bio, Shanghai, China). After ligation of the *purR* fragment and linearized vector using T4 DNA ligase (Sangon, Shanghai, China), the resulting recombinant plasmid containing *purR* was introduced into *E. coli* DH5α. Subsequently, the recombinant plasmid was extracted and transferred into *E. coli* BL21(DE3), with the transformed strain designated as BL21(DE3)-pET28a (+)-*purR*.

For protein expression, BL21(DE3)-pET28a (+)-*purR* was cultured until reaching an OD_600_ of 0.6–1.0. Then, lactose induction (4 mM) was performed, and the culture was incubated at 16 °C under low-speed shaking for more than 12 h. The bacterial pellet was resuspended in a solution containing 300 mM NaCl, 50 mM NaH_2_PO_4_, and 10 mM imidazole, and pH adjusted to 7.0. Ultrasonication was used to disrupt the cells, and the supernatant was obtained via centrifugation at 10,000× *g* for 15 min. PurR protein in the supernatant was purified using a Ni-NTA resin column, and its presence was confirmed with SDS-PAGE and Western Blotting. After desalting using G25 rapid desalting column (Bersee, Beijing, China), a final concentration of 2.0 mg/mL PurR was obtained.

### 2.11. Motif Prediction of PurR

Six genes (*purH*, *purE*, *purT*, *purL*, *purF*, *purM*) were chosen from the *pur* operon based on findings from RNA-seq analysis and the existing literature. The promoter regions of these genes were used for PurR motif prediction using the online MEME website to identify potential DNA binding sites [22]. The predicted motif was then matched with the promoter regions of the *Y. pestis* genome using the FIMO module of the online MEME website to determine the genes that can be bound by PurR in the promoter region [23].

### 2.12. Electrophoretic Mobility Shift Assay (EMSA)

Genes with significant transcriptional changes and a high FIMO matching score were selected for EMSA analysis. Fragments containing the motif region or 500 bp upstream of the start codon were used as probes in EMSA. The probes were labeled using the EMSA Probe Biotin Labelling Kit (Beyotime Biotech. Inc., Shanghai, China). After obtaining the double-stranded probe, it was denatured to single-stranded probe at 95 °C for 5 min. The labeling system was prepared following the manufacturer’s instructions, and biotin was added to the 3′ end by incubating at 37 °C for 30 min. The labeled probes were then mixed with chloroform–isopentanol (24:1) and gradually cooled to allow the single strands to reanneal into labeled double-stranded probes. Subsequently, 1/4 volume of 5 M ammonium acetate and 2 times the volume of anhydrous ethanol were added, and the mixture was precipitated at −20 °C overnight. After centrifugation and resuspension, purified labeled probes were obtained.

EMSA was performed using the Light Shift Chemiluminescent EMSA Kit (Beyotime Biotech. Inc., Shanghai, China). The EMSA binding reaction system was prepared according to the manufacturer’s protocol and experimental demands, with gentle mixing at each step, and allowed to bind at room temperature for 30 min. Following this, loading buffer was added. Low-voltage electrophoresis was then performed in 0.5 × TBE buffer until the bromophenol blue dye migrated to approximately 2/3 to 3/4 of the gel length. Subsequently, the gel was transferred onto a nylon membrane with a positive charge, and cross-linked under UV light for 20 min. The membrane was then incubated at room temperature for 15 min in blocking buffer for blocking, followed by a 30 min reaction with conjugate/blocking buffer. The membrane was washed four times with wash buffer, gently agitated for 5 min in substrate equilibration buffer, and then incubated with substrate solution for visualization. Finally, the membrane was exposed and photographed. A negative control protein was the F1 antigen of *Y. pestis*, which is a non-transcriptional regulatory factor, and a labeled segment of 16 s rRNA gene served as a negative labeled probe in this experiment. The concentrations of PurR protein and targeted DNA fragments are listed in Supplementary Table S2.

### 2.13. Reverse Transcription PCR (RT-PCR)

Gene clusters exhibiting similar transcriptional changes were identified based on the RNA-seq results. Primers were designed to amplify the intergenic regions adjacent to two genes that were potentially part of the same operon. The DNA extraction procedure followed the protocol described in Section 2.2, while the RNA extraction procedures and reverse transcription of RNA were carried out following the protocol described in Section 2.9. The obtained DNA, RNA, and cDNA were utilized as templates for PCR amplification of the predicted operon intergenic regions, with deionized water serving as a negative control. Agarose gel electrophoresis was then employed to confirm the presence of the predicted operon intergenic sections in the amplified products.

### 2.14. Ethics Statement

All animal experiments adhered to the ethical guidelines for laboratory animals in China and were conducted in accordance with the regulations outlined in laboratory animal permit no. SCXK (Jing) 2021-0006, obtained from Beijing Vital River Laboratory Animal Technology Co., Ltd. (Beijing, China). The study was approved by the Institutional Review Board at the Beijing Institute of Microbiology and Epidemiology (IACUC-IME-2023-001).

### 2.15. Statistical Analysis

The mean and variation of each of the three experimental groups were computed from three independent experiments. A *t*-test was employed to evaluate differences in the data, assuming the prerequisites of normal distribution and homogenous variance were fulfilled. When data diverged from a normal distribution, a nonparametric analysis was conducted. A one-way analysis of variance was performed, and the Student–Newman–Keuls q test was applied for multiple comparisons. Evaluation of survival curves employed the Mantel–Cox test, emphasizing the utilization of log-rank analysis. Statistical significance was set as follows: * *p* < 0.05, ** *p* < 0.01, *** *p* < 0.001, **** *p* < 0.0001.

### 2.16. Data Availability Statement

The RNA-seq data generated and analyzed in this study have been deposited at the National Microbiology Data Center under the accession numbers of NMDC40041563-40041574 (https://nmdc.cn/resource/genomics/sra/detail/NMDC40041563, accessed on 7 November 2023).

## 3. Results

### 3.1. Deletion of the PurR Makes No Difference in Growth of Y. pestis

**Figure 1:**
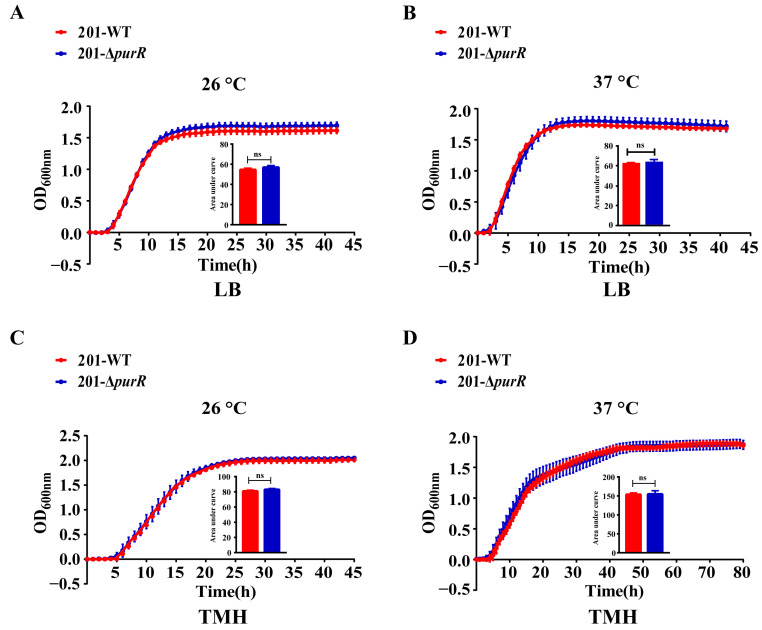
The growth curves of 201-WT and 201-Δ*purR.* The growth curves of 201-WT and 201-Δ*purR* were assessed under different culture conditions. The conditions included: (**A**) growth at 26 °C in LB medium, (**B**) growth at 37 °C in LB medium, (**C**) growth at 26 °C in TMH medium, and (**D**) growth at 37 °C in TMH medium. The bar graph presented below the growth curves illustrates the cumulative areas under the curves and is applied to statistical analysis. Each experiment included three independent biological replicates, and the results were expressed as mean ± standard deviation from three independent experiments. ns: not statistically significant.

To assess the contribution of *purR* to the growth capacity of *Y. pestis*, a comparative analysis of growth curves was performed for both strains in LB and TMH media. The findings indicated no significant differences in growth rate between these strains at temperatures of 26 °C or 37 °C. Moreover, the deletion of *purR* had no discernible effect on the growth of *Y. pestis* under two nutritional conditions, including LB medium and the nutrient-limited TMH medium (Figure 1).

### 3.2. No Differences Were Observed in the In Vitro Phenotypes of 201-WT and 201-ΔPurR

**Figure 2:**
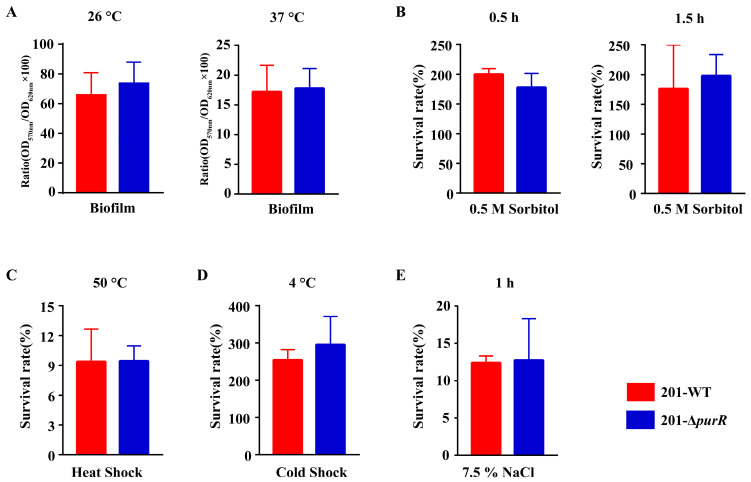
The in vitro phenotypes of 201-WT and 201-Δ*purR.* Biofilm formation of 201-WT and 201-Δ*purR* and a comparison of their survival rates in vitro under different simulated stress environments were assessed. (**A**) 0.1% crystal violet solution was used to quantify the relative amount of biofilm formation for both strains cultured at 26 °C or 37 °C (**B**). The survival rates of both strains after being stimulated by high osmotic pressure environment simulated by 0.5 M sorbitol after 30 min and after 1.5 h were compared. The survival rates of both strains after being stimulated by heat shock at 50 °C for 0.5 h (**C**) and cold shock at 4 °C for 24 h (**D**) were compared. (**E**) The survival rates of both strains after being stimulated by high salt environment simulated by 7.5% NaCl were compared after 1 h of stimulation. There were no significant differences in all results between 201-WT and 201-Δ*purR*. Each experiment was independently replicated three times for both strains, and statistical analysis was performed using a two-sample *t*-test for each comparison.

Biofilm formation plays a crucial role in the dissemination of *Y. pestis* by fleas. To investigate this phenomenon, we utilized crystal violet staining to evaluate biofilm formation in both 201-WT and 201-Δ*purR* strains. The results revealed no significant disparities in biofilm formation between the two strains at either 26 °C or 37 °C (Figure 2A). In order to simulate the environmental stresses that *Y. pestis* may encounter in natural environments, in this study, we exposed 201-WT and 201-Δ*purR* to various stressful in vitro conditions, including hypersaline, hypertonic, heat shock, and cold shock. The results demonstrated that there were no significant differences in survivability between 201-WT and 201-Δ*purR* under these environmental stresses (Figure 2B–E). These findings suggest that *purR* may not be critical for *Y. pestis* to withstand the simulated stressful environments.

### 3.3. Deletion of the PurR Attenuated the Cytotoxicity to HeLa and 293 Cells of Y. pestis

**Figure 3:**
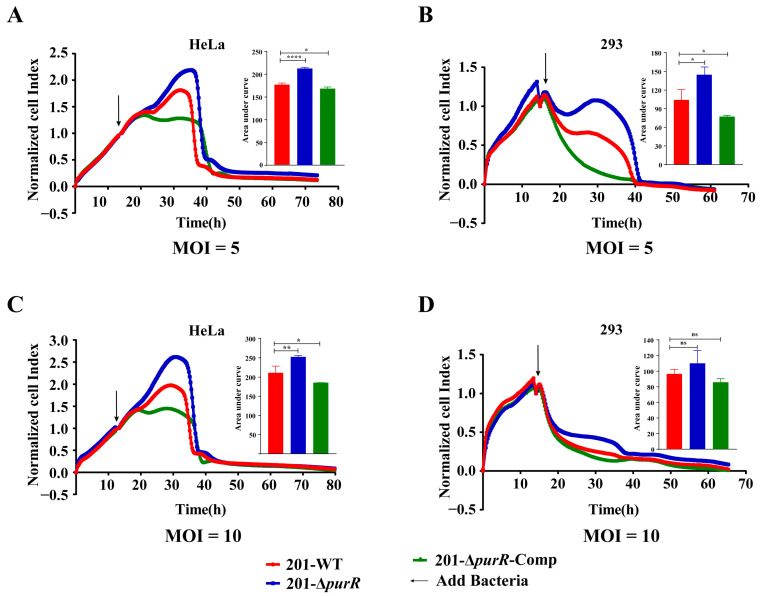
201. *purR* showed cytotoxicity attenuation on HeLa cells and 293 cells. Cells were infected with 201-WT, 201-Δ*purR*, and 201-Δ*purR*-Comp at a specific multiplicity of infection (MOI). (**A**) Bacterial infection of HeLa cells at an MOI of 5. (**B**) Bacterial infection of 293 cells at an MOI of 5. (**C**) Bacterial infection of HeLa cells at an MOI of 10. (**D**) Bacterial infection of 293 cells at an MOI of 10. The cell index was measured every 15 min. The bar graph presented next to the curves illustrates the cumulative areas under the curves and is applied to statistical analysis. At the same time point, a larger “Normalized cell index” indicates more cells or a better cell status. The higher the curve, the larger the area under the curve, indicating that the bacterial strain has weaker cytotoxicity to the cells. Each experiment included three independent biological replicates, and the results were expressed as mean ± standard deviation from three independent experiments. * *p* < 0.05, ** *p* < 0.01, **** *p* < 0.0001, ns: not statistically significant.

RTCA was used to investigate any differences in the cytotoxicity of 201-WT and 201-Δ*purR* on HeLa or 293 cells. The cell index indicated that 201-Δ*purR* exhibited significantly lower cytotoxicity to cells compared to the 201-WT strain after 10 h of stimulation. These findings were further supported by the RTCA results for 201-Δ*purR*-Comp (Figure 3). The knockout of *purR* was found to weaken the cytotoxicity of *Y. pestis* strain 201 towards HeLa and 293 cells. However, the survival curve analysis suggested that both 201-WT and 201-Δ*purR* strains exhibited similar levels of virulence when tested on mice (Supplementary Figure S1).

### 3.4. Deletion of the PurR Significantly Alters Gene Expressions in Y. pestis

In many bacteria, the main identified role of PurR is a transcriptional repressor of purine biosynthesis [6]. In this study, we conducted RNA-seq analysis of 201-Δ*purR* to identify genes potentially associated with *purR* in *Y. pestis* strain 201. Differentially expressed genes were selected based on the criteria |log_2_ (fold change) | > 0 and *p*-adjust < 0.05. Comparing these genes with 201-WT, we found significant enrichment in pathways like ribosome, carbon metabolism, and others at 26 °C (Supplementary Figure S2A) and in pathways such as carbon metabolism, purine metabolism, sulfur metabolism, ABC transport, and other metabolic processes at 37 °C (Supplementary Figure S2B).

**Figure 4:**
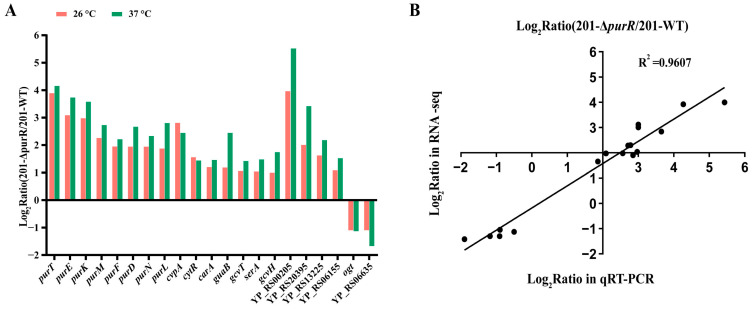
Common differentially expressed genes of 201-Δ*purR* cultured at 26 °C and 37 °C. The transcriptional level of 201-Δ*purR* was analyzed using RNA-seq and qRT-PCR under 26 °C and 37 °C culture conditions. (**A**) The 21 up-regulated or down-regulated genes that were shared under 26 °C and 37 °C culture conditions, screened with the criterion of |log_2_(FoldChange)| > 1.0; all the selected genes had a *p*-adjust value of < 10^−5^. (**B**) The correlation analysis between the 17 genes selected for qRT-PCR and these same 17 genes in the RNA-seq under 26 °C culture conditions, and the figure took point (2,2) as the origin. The selected genes were listed in the Section 2.9.

The RNA-seq results showed the up-regulation of genes involved in purine biosynthesis, including the *pur* operon, *guaB*, and *carA*, consistent with the role of *purR* as a purine repressor (Figure 4A, Table 2 and Table 3). Additionally, genes indirectly involved in purine biosynthesis, such as glycine cleavage system genes (YP_RS13225, *gcvT*, *gcvH*), were also up-regulated. However, yersiniabactin (Ybt) siderophore-related genes, which are crucial for iron uptake and *Y. pestis* virulence, such as *ybtU* [24], were down-regulated (Supplementary Tables S3 and S4). At 26 °C, the type VI secretion system (T6SS) was up-regulated (Table 2), while genes encoding peroxidase and cytochrome (*katG*, *cybB*, and *cybC*) were down-regulated (Supplementary Table S3). At 37 °C, genes involved in sulfur metabolism (*ssuB*, *ssuC*, and *ssuD*) and taurine ABC transport permeases (*tauA*, *tauB*, and *tauC*) were additionally down-regulated (Table 3). Furthermore, among the up-regulated genes, *ppsA*, encoding phosphoenolpyruvate synthase, and *cytR*, involved in DNA transcription, were included (Supplementary Table S4).

Our investigation revealed that PurR regulates purine biosynthesis in *Y. pestis* as in other bacteria, but it may also have additional functions. To validate the RNA-seq data, we selected five down-regulated genes and twelve up-regulated genes for confirmation using qRT-PCR, and the results supported the findings of RNA-seq (Figure 4B).

### 3.5. Motif Prediction of PurR

**Figure 5:**
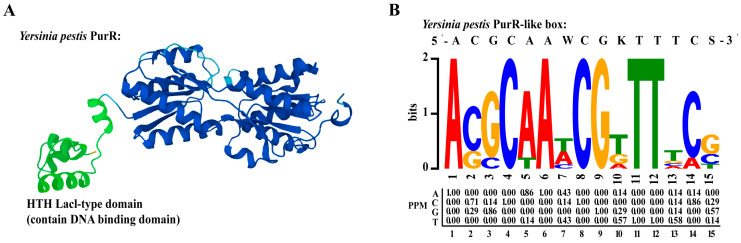
PurR structure and prediction of PurR motif. (**A**) The structure of *Y. pestis* PurR on Uniprot. Green color is the HTH lacl-type domain which contains DNA-binding domain in the front of PurR. (**B**) PurR motif predicted on the online MEME website, based on the promoter region of six genes (*purH*, *purE*, *purT*, *purL*, *purF*, *purM*) on the *pur* operon.

Transcriptional regulators are DNA-binding proteins that can modulate gene transcription by interacting with specific promoter regions. To identify the DNA-binding domain of PurR, we conducted a search for the protein structure of *Y. pestis* PurR in Uniprot and identified an HTH (helix-turn-helix) domain at the N-terminal of PurR, known for its ability to bind to DNA (Figure 5A). HTH is a common DNA-binding motif found in prokaryotic transcription factors, consisting of a short chain connecting two helices in the structure [25].

A motif is a specific DNA sequence that transcriptional regulators can recognize and bind to. In our study, we employed the online MEME tool to predict a 15-bp motif (5′-ACGCAAWCGKTTTCS-3′) for PurR (Figure 5B) [22], which exhibited high similarity to motifs previously predicted in other studies [26]. Notably, the PurR motif in *Y. pestis* was found to be similar to that of *E. coli* [7]. This observation might be attributed to the significant homology between PurR in *Y. pestis* strain 201 and PurR in *E. coli*, as the two share 82.4% identical amino acids. To identify potential promoter regions where PurR could bind, we conducted FIMO analysis on the complete promoter regions of the *Y. pestis* genome using this motif [23]. By employing a screening threshold of a *p*-value ≤ 1.0 × 10^−4^, we predicted that the promoter regions of 486 genes in *Y. pestis* strain 201 contain a motif that closely resembles a specific sequence pattern (Supplementary Table S5).

### 3.6. PurR Regulates Potential Operons in Y. pestis Strain 201

**Figure 6:**
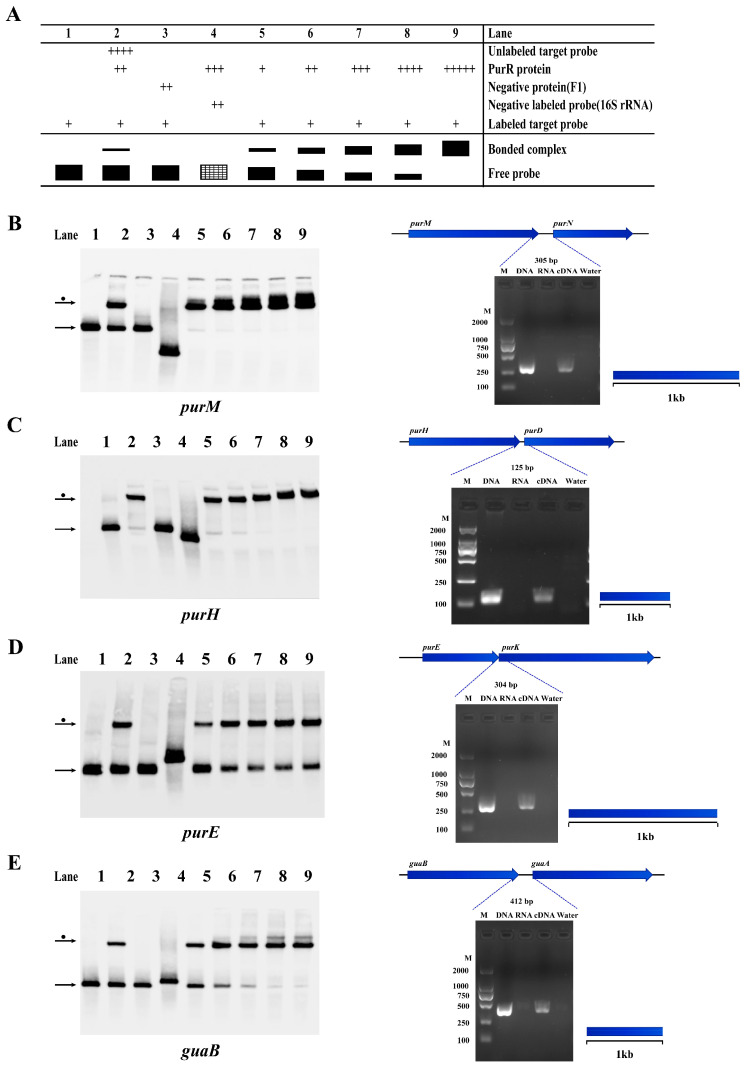
PurR may regulate some operons involved in purine biosynthesis of *Y. pestis* strain 201. The results of EMSA and RT-PCR experiments confirm the presence of operons and the regulation by PurR in *Y. pestis* strain 201, involving genes related to purine biosynthesis. (**A**) The figure illustrates the experimental setup and schematic representation of each lane in the EMSA experiment. The filled black color represents the result of the experimental group, and the shaded grid indicates the result of the negative control group. The concentrations of the components added in each experimental channel are shown in Supplementary Table S2. The width of the bands reflects the quantity of binding between the tracer probe and PurR, while the number of ‘+’ signs corresponds to the amount of the respective samples added. The identified operons include: (**B**) *purM-purN*; (**C**) *purH-purD*; (**D**) *purE-purK*; and (**E**) *guaB-guaA*. The left figure displays the EMSA results, while the right figure presents the RT-PCR results. In the RT-PCR results, the template for each gene intergenic region is indicated as DNA, RNA, cDNA, or water. The blue dotted line indicates the expected amplification fragment size for each gene intergenic region.

As mentioned previously, this study confirms that PurR can regulate purine biosynthesis by binding to the promoter region of relevant genes in *Y. pestis*. Our findings demonstrate that several PurR-regulated genes involved in purine biosynthesis are co-transcribed. For example, the *purM*-*purN*, *purH*-*purD*, and *purE*-*purK* loci are part of the *pur* operon and contribute to IMP synthesis [27,28]. Additionally, *guaB*-*guaA* participates in the conversion of IMP to GMP and AMP (Figure 6). The *carA* gene, co-transcribed with *carB* (Supplementary Figure S3B), provides arginine and uracil, which are essential for bacterial growth [29,30]. Another gene, *gcvT*, encodes glycine lyase and is part of the GCV operon, which consists of *gcvT*, *gcvH*, and *gcvP* (Supplementary Figure S3C) [31].

In addition to regulating the co-transcribed genes involved in purine biosynthesis, PurR was found to regulate two other operons, as confirmed in this study. This suggests that PurR may have additional roles in *Y. pestis* strain 201, consistent with previous findings of PurR having multiple functions in various bacterial species [12,32]. PurR was observed to interact with the promoter regions of *ssuE* and *katG*, which belong to the sulfur-starvation utilization (*ssu*) and *katG*-*cybC*-*cybB* operons [26,33], indicating that PurR could function as a regulator for these genes (Supplementary Figure S3D,E). In summary, these results imply that PurR may influence *Y. pestis* sulfur metabolism and suggest a connection between *purR* and *Y. pestis* energy metabolism.

### 3.7. PurR May Potentially Regulate Other Gene Expressions in Y. pestis Strain 201

We employed a combination of RNA-seq data and FIMO prediction to explore the potential regulatory roles of PurR in *Y. pestis*. Our findings revealed a correlation between significant transcriptional up- or down-regulation of certain genes and high matching scores in the FIMO prediction results. PurR was found to bind to the promoter regions of several other genes in the microorganism, including *pyrD* that can interact with PurR in other bacteria and contribute to the regulation of pyrimidine biosynthesis (Supplementary Figure S4C) [7]. Furthermore, PurR also exhibits self-regulation, adding an extra layer of security to its regulatory system (Supplementary Figure S4B).

Furthermore, our investigation unveiled previously unknown promoter regions of additional genes (*serA*, *ogt*, *fur*, *ybtA*, *djlA*) that PurR may bind to in *Y. pestis* strain 201 (Supplementary Figure S4D–H). Additionally, the results of EMSA and RT-PCR supported the presence of the *ybt* operon (*irp*2-*irp*1-*ybtU*-*ybtT*-*ybtE*) located on the high pathogenicity island (HPI) of *Y. pestis* (Supplementary Figure S5). These findings suggest that PurR might regulate various biological functions of *Y. pestis* beyond purine biosynthesis.

### 3.8. Potential Operons of Y. pestis

**Figure 7:**
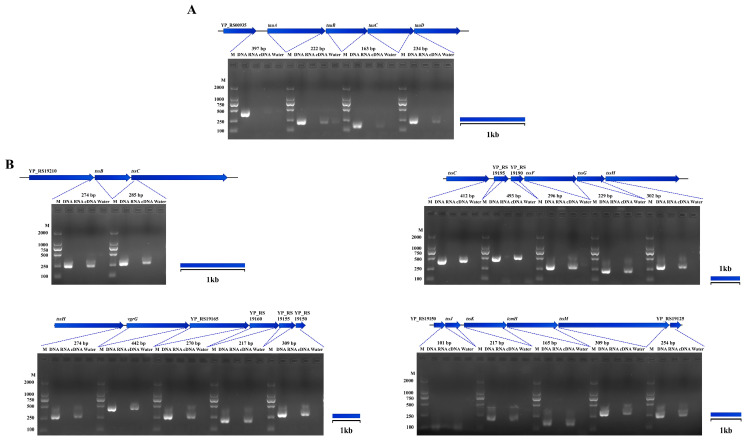
Other operon of *Y. pestis* strain 201. The RT-PCR results revealed the co-transcription of specific genes in the genome of *Y. pestis* strain 201, which was supported with RNA-seq analysis (excluding previously mentioned genes). The co-transcribed regions identified include: (**A**) YP_RS00935-*tauA-tauB-tauC-tauD* and (**B**) the type VI secretion system (T6SS).

We performed an analysis of genes that potentially undergo co-transcription based on RNA-seq data. Our investigation confirmed the presence of two potential operons in *Y. pestis*, i.e., the T6SS gene cluster and YP_RS00935-*tauA*-*tauB*-*tauC*-*tauD* (Figure 7). Similar to the *ssu* operon, the *tauABCD* operon is involved in sulfur biosynthesis in *E. coli* [34]. The T6SS is a versatile secretion system observed in various Gram-negative bacteria, and it plays a role in multiple physiological functions in *Yersinia*, including host infection, bacterial competition, and stress responses [35,36]. These findings provide evidence for the first time that T6SS undergoes co-transcription in *Y. pestis*.

## 4. Discussion

*Y. pestis*, as a multi-host pathogen, demonstrates remarkable adaptability to various environmental changes throughout its life cycle, allowing it to survive in nature, transmitted by fleas, and propagate within hosts. *Y. pestis* encounters stressful conditions, including decreased temperature, acidity, and hyperosmotic environments in nature and within fleas. Thus, we hypothesis that the loss of *purR* might plays a role in the fitness of *Y. pestis*.

As there is no significant variation in the growth rates of 201-WT and 201-Δ*purR* grown in different culture media (LB or TMH) at different temperatures (26 °C or 37 °C), it is reasonable to exclude the influence of strain growth rates in the other phenotypic tests (Figure 1). However, our study revealed that *purR* does not play a central role in *Y. pestis*’ ability to adapt to tested stressful conditions in vitro (Figure 2), even though certain genes involved in the adaptation of *Y. pestis* to harsh environments showed significant changes in transcription levels for the 201-Δ*purR* mutant (Table 2). An effective transfer approach of *Y. pestis* necessitates the production of biofilms to facilitate its transmission from fleas to mammals [37], while the results showed that the loss of *purR* makes no difference in the formation of biofilms of *Y. pestis.* Data from the multi-omics online database for *Yersinia* suggest that the transcription of *purR* remains relatively stable in *Y. pestis* [38]. Interestingly, the knockout of *purR* did not impact the growth of *Y. pestis* 201 at different temperatures and nutritional levels, consistent with findings in *S. aureus* [39]. These suggest that the transcriptional regulation of *PurR* alone may not be sufficient to disrupt the adaptive mechanism of *Y. pestis*. As mature mechanisms are often complementary and mutually reinforcing, with each factor playing a distinct role, any gaps in the regulatory system may be supplemented by other aspects.

In our study, the knockout of *purR* resulted in reduced cytotoxicity of *Y. pestis* towards HeLa and 293 cells. However, the absence of *purR* did not affect the toxicity of *Y. pestis* in mice. Due to the complexity of the infection progress, the performance of bacteria in vivo and in vitro may be inconsistent. The result in cytotoxicity of 201-Δ*purR* stands in contrast to the findings in *S. aureus*, in which the virulence is increased after the deletion of *purR*, as the mutant expresses more virulence effectors [27], whereas the expression of virulence-related Ybt siderophore-related genes decreased in the *Y. pestis purR* knockout strains. We only performed preliminary animal challenge experiments in this study; it can be determined if the knockout of *purR* affects the toxicity of *Y. pestis* to animals based on the bacterial load and pathological changes of specific organs in a follow up study.

Previous research has identified the PurR protein as an HTH-type transcriptional repressor in bacteria, and has already confirmed its role in regulating purine biosynthesis by repressing the *pur* operon in other bacterial species [6,40,41]. In this study, we examined the purine regulatory function of PurR in *Y. pestis* 201. Our findings revealed that the absence of *purR* led to an upregulation of purine biosynthesis-related genes and that PurR exhibited binding affinity to the promoter region of nearly all these genes. Furthermore, we also identified eleven potential operons in *Y. pestis* 201 through RT-PCR analysis, eight of which could be bound by PurR, highlighting the role of PurR as a regulator of multiple operons and thereby establishing a potential regulon. Interestingly, PurR was shown to bind to the promoter region of *fur*, the gene encoding ferric uptake regulator Fur in *Y. pestis*, which was also found to regulate siderophore-associated operons including the *ybt* operon [42], indicating a cross-regulation of transcriptional regulators. This interconnection creates a vast and complex regulatory network for PurR in *Y. pestis.*

In addition to its role in regulating purine biosynthesis, we sought to explore other potential functions of *purR* in *Y. pestis*. Our analysis revealed that *purR* may also influence the virulence, sulfur metabolism, and energy synthesis of *Y. pestis* by directly or indirectly regulating specific operons, i.e., *irp2*-*irp1*-*ybtU*-*ybtT*-*ybtE*, *ssuE*-YP_RS20420-*ssuD*-*ssuC*-*ssuB*, and *katG*-*cybC*-*cybB*, respectively. Notably, we discovered that the T6SS (Type VI Secretion System) gene cluster was co-transcribed in *Y. pestis* 201, and the knockout of *purR* resulted in an increase in transcription of these genes. T6SS has been shown to play a crucial role in the interaction between *Y. pestis* and macrophages [43]. This finding provides valuable insights for studying the mechanism of interaction between *Y. pestis* and macrophages.

In summary, this study conducted a preliminary investigation into the function of *purR* in *Y. pestis* 201 and provided an initial analysis of the regulatory network of PurR. The findings laid the groundwork for future research, but a more comprehensive understanding of the underlying mechanism requires additional methods and robust evidence for validation.

## Figures and Tables

**Table 1:** Bacterial strains and plasmids used in this study.

Strain or Plasmid	Genotype	Reference
*E. coli*		
S17λ*pir*	Tp^r^ Sm^r^ *recA thi pro hsdR^−^M^+^* (RP4-2-Tc::Mu: Kan^r^Tn7) *λpir*	[17]
S17-pDS132-*purR*-del	pDS132-*purR*-del was introduced into S17λpir	This study
DH5α	F- φ80lacZΔM15 Δ(*lacZYA*-arg F) U169 *endA*1 *recA*1 *hsdR*17(rk−, mk+) *supE*44 *λ*- thi-1 *gyrA*96 *relA*1 *phoA*	[17]
DH5α-pACYC184- *purR*	pACYC184-*purR* was introduced into DH5α	This study
DH5α-pET28a (+)-*purR*	pET28a (+)-*purR* was introduced into DH5α	This study
BL21(DE3)	F-ompT *hsdSB*(r_B_^−^m_B_^−^) gal dcm (DE3)	[18]
BL21(DE3)-pET28a (+)-*purR*	pET28a (+)-*purR* was introduced into BL21 (DE3)	This study
*Y. pestis*		
201-WT	*Y. pestis* biovar Microtus strain 201, WT	[14]
201-Δ*purR*	deleted *purR* based on strain 201	This study
201-Δ*purR*-Comp	201-Δ*purR* containing plasmid pACYC184-*purR*	This study
plasmids		
pDS132	suicide vector, derived from pCVD442, without IS1 sequences. *bla* gene replaced by the *cat* gene	[17]
pACYC184	cloning vector, Cm^r^ Tet^r^	[17,19]
pET28a (+)	overexpression vectors, carry an N-terminal His-Tag/T7-Tag configuration plus an optional C-terminal His-Tag sequence, Kan^r^	[18]

**Table 2:** Differential expressions of genes associated to purine biosynthesis and T6SS in 201-Δ*purR* in comparison with 201-WT at 26 °C.

Locus Tag *	Log^2^FC	*p*-Adjust	Gene Name	Gene Description	Pathway Name
YP_RS08450	3.92	5.23 × 10^−120^	*purT*	formate-dependent phosphoribosylglycinamide formyltransferase	Purine metabolism
YP_RS04375	3.12	4.25 × 10^−46^	*purE*	5-(carboxyamino)imidazole ribonucleotide mutase	
YP_RS04370	3.01	2.65 × 10^−52^	*purK*	5-(carboxyamino)imidazole ribonucleotide synthase	
YP_RS16125	2.30	1.50 × 10^−46^	*purH*	bifunctional phosphoribosylaminoimidazolecarboxamide formyltransferase/IMP cyclohydrolase	
YP_RS14005	2.29	1.70 × 10^−55^	*purM*	phosphoribosylformylglycinamidine cyclo-ligase	
YP_RS12405	1.98	2.79 × 10^−52^	*purF*	amidophosphoribosyltransferase	
YP_RS16130	1.97	1.56 × 10^−34^	*purD*	phosphoribosylamine--glycine ligase	
YP_RS14010	1.97	1.16 × 10^−26^	*purN*	phosphoribosylglycinamide formyltransferase	
YP_RS13160	1.90	1.40 × 10^−21^	*purL*	phosphoribosylformylglycinamidine synthase	
YP_RS14225	1.21	2.03 × 10^−16^	*guaB*	IMP dehydrogenase	
YP_RS19195	1.95	3.45 × 10^−27^	-	Hcp family type VI secretion system effector	Bacterial secretion system
YP_RS19175	1.89	3.77 × 10^−24^	*tssH*	type VI secretion system ATPase TssH	
YP_RS19210	1.83	3.03 × 10^−21^	-	ImpA family type VI secretion system protein	
YP_RS19205	1.82	2.89 × 10^−25^	*tssB*	type VI secretion system contractile sheath small subunit	
YP_RS19200	1.80	2.38 × 10^−27^	*tssC*	type VI secretion system contractile sheath large subunit	
YP_RS19185	1.79	1.88 × 10^−18^	*tssF*	type VI secretion system baseplate subunit TssF	
YP_RS19145	1.78	4.53 × 10^−19^	*tssJ*	type VI secretion system lipoprotein TssJ	
YP_RS19180	1.72	8.93 × 10^−18^	*tssG*	type VI secretion system baseplate subunit TssG	
YP_RS19140	1.71	1.51 × 10^−25^	*tssK*	type VI secretion system baseplate subunit TssK	
YP_RS19135	1.68	5.57 × 10^−37^	*icmH*	type IV secretion system protein IcmH/DotU	
YP_RS19170	1.62	1.09 × 10^−15^	*vgrG*	type VI secretion system tip protein VgrG	
YP_RS19190	1.60	2.14 × 10^−18^	-	type VI secretion system baseplate subunit TssE	
YP_RS19130	1.45	3.02 × 10^−28^	*tssM*	type VI secretion system membrane subunit TssM	
YP_RS15960	1.44	2.50 × 10^−5^	*tssE*	type VI secretion system baseplate subunit TssE	
YP_RS15965	1.11	1.88 × 10^−15^	*tssC*	type VI secretion system contractile sheath large subunit	
YP_RS15970	1.03	2.09 × 10^−6^	*tssB*	type VI secretion system contractile sheath small subunit	
YP_RS15955	0.96	8.61 × 10^−4^	*tssF*	type VI secretion system baseplate subunit TssF	

*: NCBI reference sequence: ASM788v1.

**Table 3:** The genes of purine biosynthesis and sulfur metabolism system expression changes in 201-Δ*purR* in comparison with 201-WT at 37 °C.

Locus Tag *	Log^2^FC	*p*-Adjust	Gene Name	Gene Description	KEGG
YP_RS08450	4.18	1.68 × 10^−95^	*purT*	formate-dependent phosphoribosylglycinamide formyltransferase	Purine metabolism
YP_RS04375	3.76	4.12 × 10^−46^	*purE*	5-(carboxyamino)imidazole ribonucleotide mutase	
YP_RS04370	3.60	5.14 × 10^−64^	*purK*	5-(carboxyamino)imidazole ribonucleotide synthase	
YP_RS16125	2.97	1.89 × 10^−64^	*purH*	bifunctional phosphoribosylaminoimidazolecarboxamide formyltransferase/IMP cyclohydrolase	
YP_RS13160	2.83	4.37 × 10^−41^	*purL*	phosphoribosylformylglycinamidine synthase	
YP_RS14005	2.76	8.61 × 10^−66^	*purM*	phosphoribosylformylglycinamidine cyclo-ligase	
YP_RS16130	2.70	2.68 × 10^−33^	*purD*	phosphoribosylamine--glycine ligase	
YP_RS14225	2.47	3.44 × 10^−23^	*guaB*	IMP dehydrogenase	
YP_RS14010	2.36	1.28 × 10^−22^	*purN*	phosphoribosylglycinamide formyltransferase	
YP_RS12405	2.24	4.64 × 10^−27^	*purF*	amidophosphoribosyltransferase	
YP_RS13925	1.95	8.44 × 10^−11^	-	phosphoribosylaminoimidazolesuccinocarboxamide synthase	
YP_RS14215	1.75	1.04 × 10^−14^	*guaA*	glutamine-hydrolyzing GMP synthase	
YP_RS09185	1.26	1.20 × 10^−7^	*purB*	adenylosuccinate lyase	
YP_RS20415	−2.40	3.19 × 10^−8^	*ssuD*	FMNH2-dependent alkanesulfonate monooxygenase	Sulfur metabolism
YP_RS20410	−1.80	2.48 × 10^−4^	*ssuC*	aliphatic sulfonate ABC transporter permease SsuC	
YP_RS00940	−1.79	7.13 × 10^−4^	*tauA*	taurine ABC transporter substrate-binding protein	
YP_RS00950	−1.79	1.23 × 10^−3^	*tauC*	taurine ABC transporter permease TauC	
YP_RS00945	−1.59	1.27 × 10^−3^	*tauB*	taurine ABC transporter ATP-binding subunit	
YP_RS20405	−1.35	5.37 × 10^−2^	*ssuB*	aliphatic sulfonates ABC transporter ATP-binding protein	
YP_RS00425	−1.19	5.53 × 10^−2^	-	sulfate ABC transporter substrate-binding protein	

*: NCBI reference sequence: ASM788v1.

## Data Availability

All data presented in this study are available in this published article. The RNA-seq data generated and analyzed are available in the National Microbiology Data Center under the accession numbers of NMDC40041563-40041574.

## Supplementary Materials

The following supporting information can be downloaded at: https://www.mdpi.com/article/10.3390/microorganisms11112801/s1, Figure S1: 201-WT and 201-ΔpurR showed virulence no differences in mice; Figure S2: KEGG maps of the differentially expressed genes of 201-ΔpurR culture at 26 °C and at 37 °C; Figure S3: PurR may regulate other operons of Y. pestis strain 201; Figure S4: PurR may regulate some genes of Y. pestis strain 201; Figure S5: The ybt operon in Y. pestis strain 201; Table S1: Primers used in the study; Table S2: EMSA settings for different target genes; Table S3: 201-ΔpurR differentially significantly expressed gene at 26 °C; Table S4: 201-ΔpurR differentially significantly expressed gene at 37 °C; Table S5: The results of FIMO analysis.
