# Supplementary material for: Regulatory Functions of PurR in Yersinia pestis: Orchestrating Diverse Biological Activities
Source: Microorganisms. 2023 Nov 17;11(11):2801. doi: 10.3390/microorganisms11112801 (PMC10673613; doi:10.3390/microorganisms11112801)
Supplement: Supplementary file 1 [file microorganisms-11-02801-s001.zip › Supplementary File/Supplementary Figures.pdf]

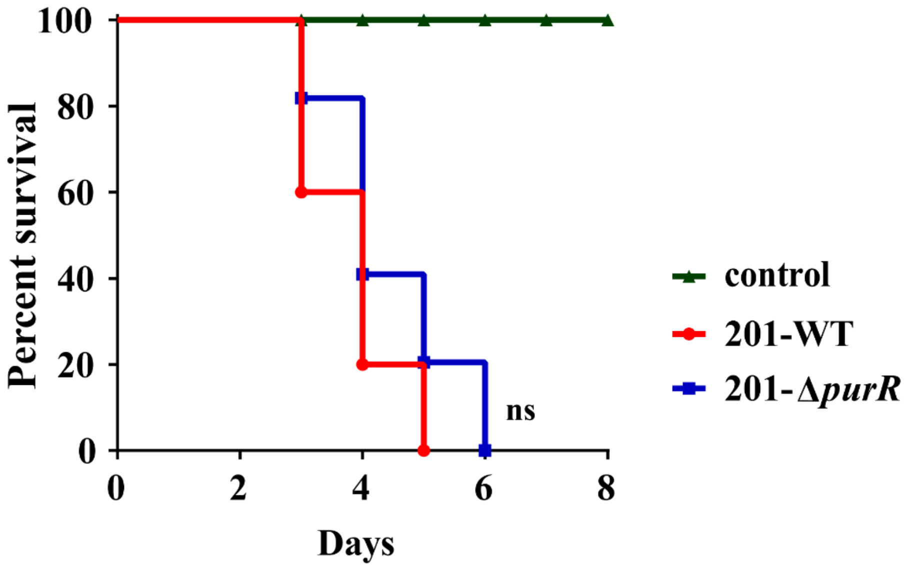

Supplementary Figure S1. 201-WT and 201-Δ*purR* showed virulence no differences in mice.

Survival curves of female BABL/c mice (8-10 weeks old) after intraperitoneal challenges with 201-WT and 201-Δ*purR* were analyzed. Each group consisted of 10 mice.

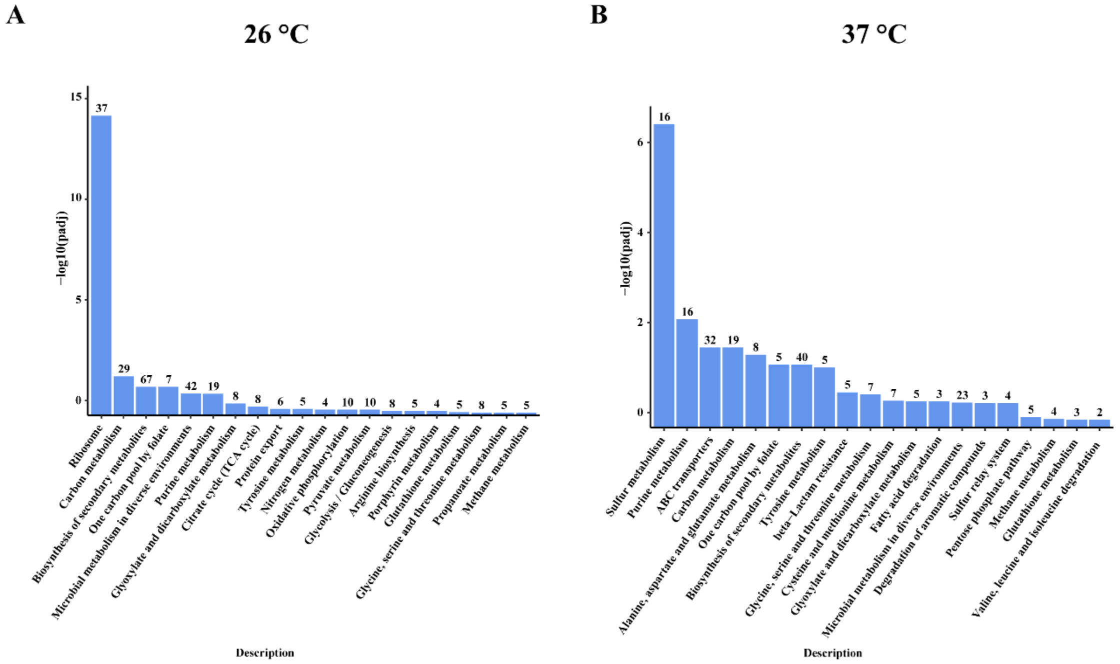

Supplementary Figure S2. KEGG maps of the differentially expressed genes of 201-Δ*purR* culture at 26°C (A) and at 37°C (B).

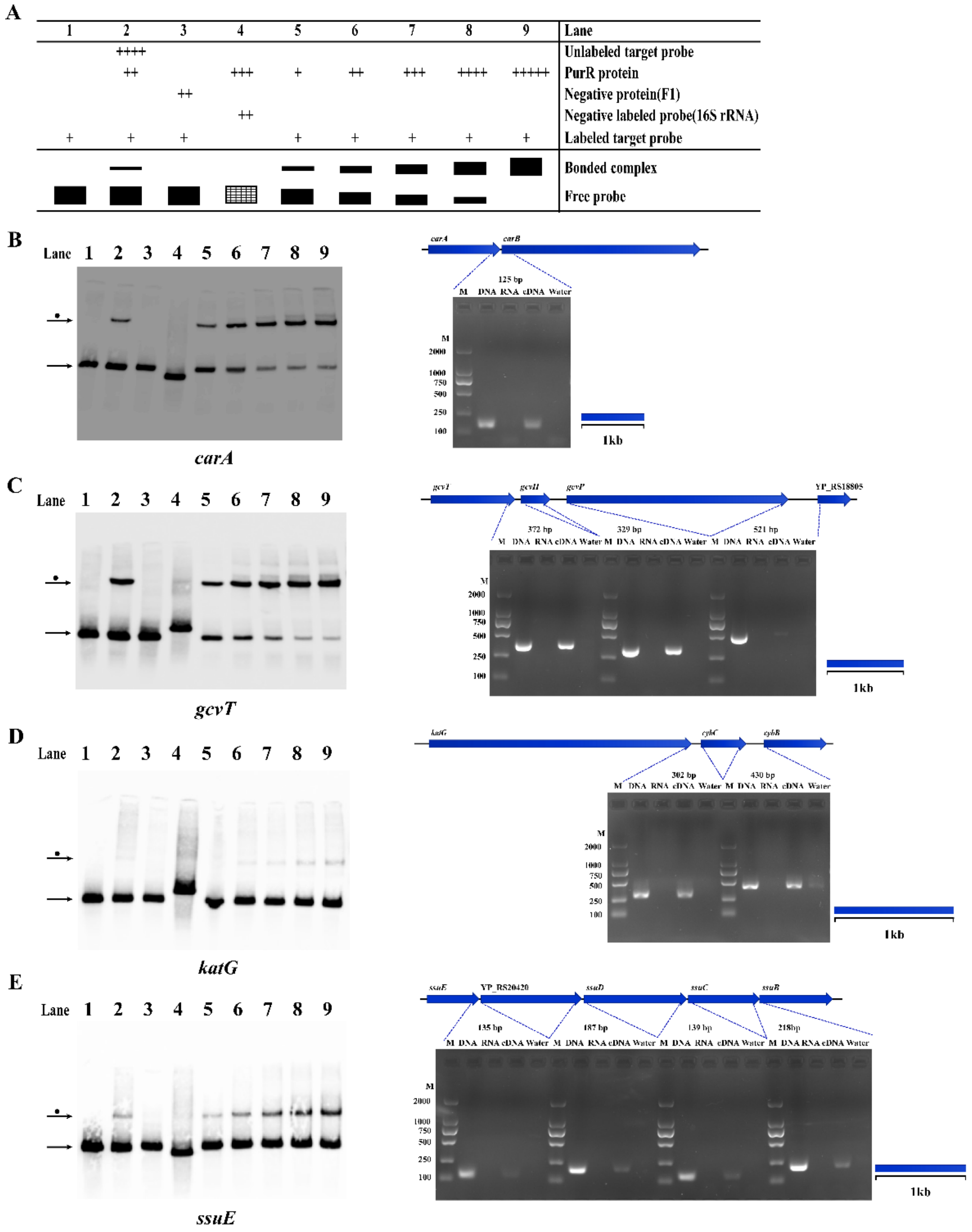

Supplementary Figure S3. PurR may regulate other operons of *Y. pestis* strain 201.

EMSA and RT-PCR experiments provide evidence of operon existence which were regulated by PurR in *Y. pestis* strain 201. (A) The experimental setup and result schematic for each lane in the EMSA experiment are presented. The filled black color corresponds to results from the experimental group, the shaded grid signifies outcomes from the negative control group. The concentrations of the components added in each experimental channel are shown in Supplementary Table 2. The identified operons encompass the following: (B) *carA-carB*; (C) *gcvT-gcvH-gcvP*-YP_RS18805; (D) *katG-cybC-cybB*; (E) *ssuE*-YP_RS20420-*ssuD-ssuC-ssuB*. The left panel illustrates the EMSA findings, while the right panel displays the RT-PCR results.

For the RT-PCR outcomes, the template utilized for each gene's intergenic region comprises DNA, RNA, cDNA, or water. The anticipated amplification fragment size of each gene's intergenic region is denoted by the blue dotted line.

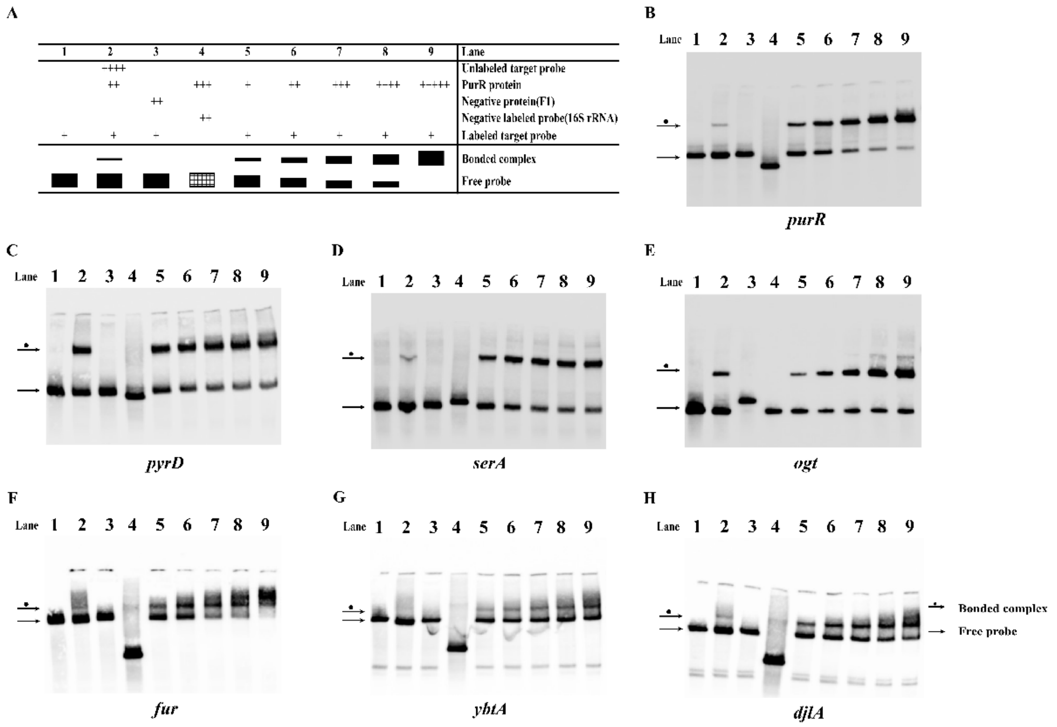

Supplementary Figure S4. PurR may regulate some genes of *Y. pestis* strain 201.

The EMSA findings provide evidence of PurR's regulatory role on selected genes within *Y. pestis* strain 201. (A) The figure illustrates the experimental configuration and schematic representation of outcomes for each lane in the EMSA experiment. The filled black color corresponds to results from the experimental group, the shaded grid signifies outcomes from the negative control group. The concentrations of the components added in each experimental channel are shown in Supplementary Table 2. The genes subject to PurR regulation are as follows: (B) *purR*; (C) *pyrD*; (D) *serA*; (E) *ogt*; (F) *fur*; (G) *ybtA*; (H) *djlA*.

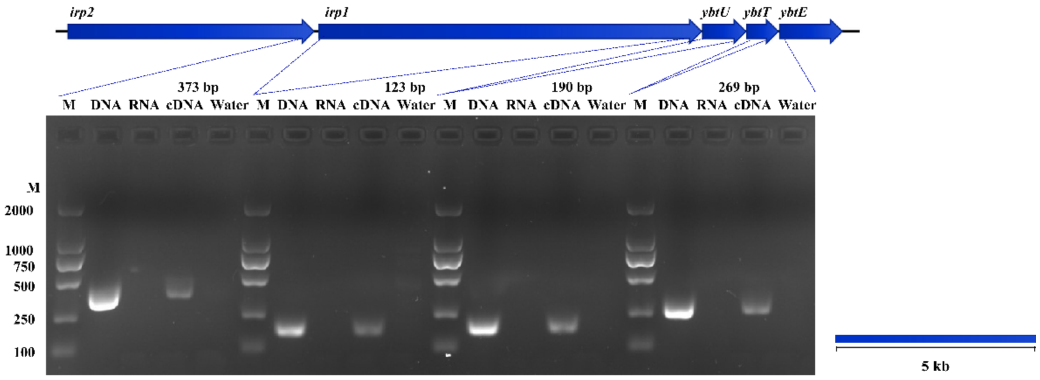

Supplementary Figure S5. The *ybt* operon in *Y. pestis* strain 201.

The RT-PCR results of the *ybt* operon in *Y. pestis* strain 201.
