# Supplementary material for: Regulatory Functions of PurR in Yersinia pestis: Orchestrating Diverse Biological Activities
Source: Microorganisms. 2023 Nov 17;11(11):2801. doi: 10.3390/microorganisms11112801 (PMC10673613; doi:10.3390/microorganisms11112801)
Supplement: Supplementary file 1 [file microorganisms-11-02801-s001.zip › Supplementary File/Supplementary Table/Supplementary Table S1.pdf]

Supplementary Table S1 Primers used in the study

Primer	Primer sequence (5'-3')	Primer function
**Construction of 201-Δ*purR***		
Pre-*purR*-F(525)	AGAGGTACCGCATGCCAAGCTACATCGATAACATA	Amplify the upstream homology arm of *purR*.
Pre-*purR*-R	ACGACCGGCCAAGGCAAGCAAAAAAGAATGACTCCTAAAC	
Post-*purR*-F(581)	TGCTTGCCTTGGCCGGTCGT	Amplify the downstream homology arm of *purR*.
Post-*purR*-R	TTCCCGGGAGAGCTCCCCAGTGGGTGGGCGTTTTG	
pDS132-F(1343)	GTTTCTGTTGCATGGGCATAAAG	Identify whether the recombinant vector is constructed successfully
pDS132-R	AACAAGCCAGGGATGTAACG	
purR-seq-F(1315)	AACTTTGGCGCTGGTTGGAC	Identify whether the *purR* is knocked out successfully.
purR-seq-R	TGGGTGGGCGTTTTGTCTTT	
purR-F(0)	GGTAGAAAATAGCTGCT	Amplify that internal sequence of *purR*, to identify whether the knockout is successful.
purR-R	TCTCTTTAGGTTGATGG	
**Construction of 201-Δ*purR*-Comp**		
184-*purR*-F(1331)	ATCATCGATAAGCTTTAGGAAAAAGGCACATA	Amplifying *purR* whole gene with enzyme cutting site.
184-*purR*-R	CGGCGTAGAGGATCCTCAACGGCGATAATCAC	
pACYC184-F(1653)	CGGTTCAAAGAGTTGGT	Identify whether pACYC184-*purR* is constructed successfully.
pACYC184-R	TGATGTCGGCGATATAG	
**Construction of PurR expression vector**		
pET28a(+)-*purR*-F(1044)	CCCAAGCTTATGGCAACGATTAAAGATGTGG	Amplifying *purR* whole gene with enzyme cutting site.
pET28a(+)-*purR*-R	CGCGGATCCTCAACGGCGATAATCACGG	
T7-F(1325)	TAATACGACTCACTATAGGG	Identify whether pET28a(+)-*purR* is constructed successfully.
T7-Ter-R	GCTAGTTATTGCTCAGCGG	

Primer	Primer sequence (5'-3')	Primer function
**qRT-PCR**		
ybtE-F(139)	GCCCTTATACCATTTCG	Amplify *ybtE* internal
ybtE-R	TCTTTTATGCGTCCCTC	sequence.
irp1-F(137)	CGGTCTGCCCATTTTAC	Amplify *irp1* internal
irp1-R	CAGCCACCTGTTTTTCC	sequence.
ybtU-F(143)	TCCACCCGGATGACATC	Amplify *ybtU* internal
ybtU-R	CTTAGCCAGGCAGCGAC	sequence.
ybtT-F(191)	CCATCTGGAACCGCTCA	Amplify *ybtT* internal
ybtT-R	GGCACCCGGAAATAATC	sequence.
purK-F(157)	CAGCAAACTACAGCAGC	Amplify *purK* internal
purK-R	TTGTGTCCAGTGACCAC	sequence.
purE-F(125)	CCTTGAGTGGCGTTGAT	Amplify *purE* internal
purE-R	TGCTAAGATTTGTGCGG	sequence.
purT-F(121)	CAGCGAGCAAAAGAGAT	Amplify *purT* internal
purT-R	GTGGGCGAGGAGAAACT	sequence.
purF-F(155)	GGTGAAGGATGTATTTG	Amplify *purF* internal
purF-R	CATTGTGAGCCAGGGTA	sequence.
cvpA-F(183)	CAGCCATTTTTACACTT	Amplify *cvpA* internal
cvpA-R	ACCCCTAACACCCTATC	sequence.
purL-F(139)	TTTGTCGGCTTTTCGTA	Amplify *purL* internal
purL-R	CTGGAGTCTGGCGTGTT	sequence.
purM-F(153)	CTGTGTGGGTGTCGTAG	Amplify *purM* internal
purM-R	TGTTCTGGATTGGTGTT	sequence.
purN-F(145)	CCACCGCCAGGCACTCG	Amplify *purN* internal
purN-R	CCGTTCCGCCACATCTT	sequence.
purH-F(123)	TGCTCGTTGGAAGATGC	Amplify *purH* internal
purH-R	AATAATGGCGGGGTAGT	sequence.
YP_RS10830-F(177)	CGTCAAAACAGCGGGTA	Amplify YP_RS10830 internal
YP_RS10830-R	TGGCAAGGAACAACAAT	sequence.
YP_RS00205-F(149)	GTTACCCGCAGAAGATA	Amplify YP_RS00205 internal
YP_RS00205-R	TCAACGGAGAAACAAAG	sequence.
YP_RS20395-F(119)	CAGCAACCCTCAAGCAC	Amplify YP_RS20395 internal
YP_RS20395-R	AAACCATCGCCAGAAAA	sequence.

Primer	Primer sequence (5'-3')	Primer function
YP_RS13225-F(133)	CAAAAACAGCGTACCTA	Amplify YP_RS13225 internal
YP_RS13225-R	CACATCACACATCCAGC	sequence.
16S-F(110)	TAGAGTCTTGTAGAGGGGGG	Amplify that internal sequence
16S-R	CCTGAGCGTCAGTCTTTGTC	of the internal reference gene 16S rRNA.
**EMSA**		
16S-EMSA-F(230)	GACAAAGACTGACGCTCAGG	Amplify negative probe
16S-EMSA-R	CGTTGCATCGAATTAAACCA	sequences.
purR-pro-F(276)	GTGTGGAATGATTTTCCCAC	Amplify the promoter region
purR-pro-R	AATGACTCCTAAACTGACCG	of *purR.*
purH-pro-F(262)	GCCCGCAACCGCTATTTTCA	Amplify the promoter region
purH-pro-R	GGAGTCGCAGTTTTCTTCAT	of *purH.*
purM-pro-F(383)	TTCTCACGCATCAAACCAAG	Amplify the promoter region
purM-pro-R	GCAATCACCCGAATCACTTA	of *purM.*
pyrD-pro-F(242)	CCGTGTTTTTACGATTTGCC	Amplify the promoter region
pyrD-pro-R	GTTACTCGGTTGCTGCCTGC	of *pyrD.*
serA-pro-F(196)	TCACATTCCCTCATTTCCTT	Amplify the promoter region
serA-pro-R	AGACTTCAGATGCGGCTGCC	of *serA.*
carA-pro-F(220)	CGATTAGTGTCTTTTTGGTT	Amplify the promoter region
carA-pro-R	TTTTCTCTATTTTTTGCCGT	of *carA.*
gcvT-pro-F(176)	CTTCTGTTTTTTGTCGCCTG	Amplify the promoter region
gcvT-pro-R	CCCTCAATATACACTTCGTG	of *gcvT.*
ogt-pro-F(160)	CGGATAAAGTAGGTACACGG	Amplify the promoter region
ogt-pro-R	TATGAAAATGAGAGGGCAAA	of *ogt.*
guaB-pro-F(190)	CTGACAGTAAAAATTGAGGA	Amplify the promoter region
guaB-pro-R	GGTGAATGTGAAAAAGATAA	of *guaB.*
ssuE-pro-F(258)	GCAAGGCGCTTGAATATTGG	Amplify the promoter region
ssuE-pro-R	TTACTGGGTGGGCTGGATGA	of *ssuE.*
purE-pro-F(148)	TCATGACGAATAACAGCGTA	Amplify the promoter region
purE-pro-R	GGCGAAGAGGGCGTAGAGCA	of *purE.*
katG-pro-F(172)	AAACGCCCTACGCCTTGTCC	Amplify the promoter region
katG-pro-R	ATGCCCCTTACCCACTGCCC	of *katG.*
fur-pro-F(638)	CGCTGAGTATTTCTGTG	Amplify the promoter region

Primer	Primer sequence (5’-3’)	Primer function
fur-pro-R	TAAGGCTTTGTTGTTGT	of *fur*.
ybtA-pro-F(548)	GCTGCCCATTTGCTGTA	Amplify the promoter region
ybtA-pro-R	GCGTTTGCGGTGACTCT	of *ybtA*.
djlA-pro-F(552)	TGCGGATCGTCGTAGTT	Amplify the promoter region
djlA-pro-R	GAGCAGTTTTCCCCAAT	of *djlA*.
**RT-PCR**		
purEK-F(304)	CCCTGATCCGAGGGAAGAAG	Amplification of the intergenic
purEK-R	CAGTTGCCAAACCCAAGCTA	region of *purE*-*purK*.
purMN-F(305)	TGGCAGATAAAGCGGTTGAG	Amplification of the intergenic
purMN-R	GATACGCCCTTGTTGTTGGG	region of *purM*-*purN*.
purHD-F(125)	TCACCGACATGCGCCATTTC	Amplification of the intergenic
purHD-R	GTTCACGACCGCCGTTACCA	region of *purH*-*purD*.
gcvTHP-F1(372)	GTGTATTTTTTTGATGCCGC	
gcvTHP-R1	CATGTTCAGTGATCCCGACG	Amplification of the intergenic
gcvTHP-F2(329)	TTGGATGCTGATGCTTACCT	region of *gcvT*-*gcvH*-*gcvP*-
gcvTHP-R2	GCTGCTGTTCAACTGAGGAG	YP_RS18805.
gcvTHP-F3(521)	GGCGGGTGTACTGGAGAATA	
gcvTHP-R3	AATGACCAACTGCCGATGAA	
tauABCD-F1(397)	GATGATAAGCCTGGGGATGT	
tauABCD-R1	AGGGTTGCCGAAGAAAAAGT	
tauABCD-F2(222)	CAGCAAGGCAAGATCCCTCA	Amplification of the intergenic
tauABCD-R2	GCCCCAAGACCACAACCAAC	region of YP_RS00935-*tauA*-
tauABCD-F3(163)	GTGAACCTTGCCGTGCTATT	*tauB*-*tauC*-*tauD*.
tauABCD-R3	CGTTTTTTTCGGTGCATCTG	
tauABCD-F4(234)	ATTGCTTTTGGGATGGAGTT	
tauABCD-R4	ACGGTGTGATCGGTTGATTA	
guaBA-F(412)	GAAAGAGATCGTGCACCAAC	Amplification of the intergenic
guaBA-R	AGCCGAAATCAAGAATAAGG	region of *guaB*-*guaA*.
carAB-F(125)	CGAACTGATTGAGGCTTACCG	Amplification of the intergenic
carAB-R	AGTAGTCAAACTCACAAGCC	region of *carA*-*carB*.
ssuEDCB-F1(135)	TTAGAAGAAGCGTTGGAAAG	Amplification of the intergenic
ssuEDCB-R1	AGAAGAAAGATAGCGACATA	region of *ssuE*-YP_RS20420-
ssuEDCB-F2(187)	AGCAAAATCACAGACCAAAT	*ssuD*-*ssuC*-*ssuB*.

**Primer**	**Primer sequence (5’-3’)**	**Primer function**
ssuEDCB-R2	GATCAACACACCACCGAACC	
ssuEDCB-F3(139)	GCCACAAAAAGTCTCGCAAA	
ssuEDCB-R3	ATAACCAACCCGCCTCAACC	
ssuEDCB-F4(218)	TCGGGTATTGGTTATCTGGC	
ssuEDCB-R4	CGTTATTGGCGTCCCTTGCG	
katGCB-F1(302)	AACAGGGAAACTTAAATGGA	
katGCB-R1	GATGAGTAATGAGAAAACGC	Amplification of the intergenic region of *katG*-*cybC*-*cybB*.
katGCB-F2(430)	GAGGTAAAAAAATCGGTTGG	
katGCB-R2	GTACTATTGGTGGTGTCGGG	
ybtUTE-F1(373)	TGACCTCTTCACCCACCCTT	
ybtUTE-R1	GCATTCACGACCTTCCAGCA	
ybtUTE-F2(123)	CCCACGTTCAGGCTTGTGCG	Amplification of the intergenic region of *irp2*-*irp1*-*ybtU*-*ybtT*-*ybtE*.
ybtUTE-R2	GACCAGTTCCAGCCCCTCCG	
ybtUTE-F3(190)	TCCACCAGCGGCCTGCCAAA	
ybtUTE-R3	GCCACAGCGGGATGCACATT	
ybtUTE-F4(269)	GTCCGCCATTTTCCCCACGC	
ybtUTE-R4	TCCCCCGAACGCAAACCCAG	
T6SS-F1(274)	CATTCGCCGTACCAAAGAGA	
T6SS-R1	CCAGAAAGATCAGCCATCAC	
T6SS-F2(285)	CTTGAACAACCTGATTTTTT	
T6SS-R2	CTTGGCCTTTATTGGCATAC	
T6SS-F3(412)	TTTACGCCCTCACTACCAAC	
T6SS-R3	GACTTTCCCAGAACCACCAC	
T6SS-F4(493)	TCAGGTCAAGGTCAACAAGA	
T6SS-R4	TACAGAAGCCTCACAATGCG	Amplification of the intergenic region of T6SS cluster.
T6SS-F5(296)	CGGGCGAAGAGCACTTGGTC	
T6SS-R5	AACGCTGTTGGATACGGGAA	
T6SS-F6(229)	TTCAACAGCAGCCACCACAG	
T6SS-R6	ACGCAGCAACTCGAAAAAGT	
T6SS-F7(302)	AATCATACAGACCTCACCCA	
T6SS-R7	AGTAGAGCAGGAATATCCCC	
T6SS-F8(274)	GCTGTTGGCAATGAAGTGTT	
T6SS-R8	CGTCTTGAAGGAGAGAGGAA	

Primer	Primer sequence (5'-3')	Primer function
T6SS-F9(442)	CAGTATTGGGACGGTAGGAA	
T6SS-R9	CTCTGCACTCAGCGAGGTAA	
T6SS-F10(270)	GGCGATGATCAGCCAAAACA	
T6SS-R10	CAATCTCGGCAAACGAAATA	
T6SS-F11(217)	GGTCCAAAAAATAACCCTAA	
T6SS-R11	TGTTTCATACCCGAGCAGTG	
T6SS-F12(309)	TTCGTGAGAGCCAAACAAGC	
T6SS-R12	TACCCCCACAGCAAAAATAG	
T6SS-F13(101)	ATGACCAATAATTACAACGC	
T6SS-R13	GGATAAAAATAGGCAACACA	
T6SS-F14(217)	GGTGCTCGTTGATGGTAAGG	
T6SS-R14	GTTGTTGAAAATGCTGGGGT	
T6SS-F15(165)	TTCCCTGAGTTGCGTTTTGA	
T6SS-R15	AGGCTACGGTGCTTTTGGCT	
T6SS-F16(309)	TGACAATGCAACGGAAGAAG	
T6SS-R16	CCACAGTGCCCAAAATAAAA	
T6SS-F17(254)	CAAAAGCATTGGCACAGTTC	
T6SS-R17	CTTATTCATATCGCTCATCA	

The underlined bases indicate the restriction enzyme sites.

The digit of () indicate expected fragment size for the primers amplification, and unit is bp.
