# Supplementary material for: Regulatory Functions of PurR in Yersinia pestis: Orchestrating Diverse Biological Activities
Source: Microorganisms. 2023 Nov 17;11(11):2801. doi: 10.3390/microorganisms11112801 (PMC10673613; doi:10.3390/microorganisms11112801)
Supplement: Supplementary file 1 [file microorganisms-11-02801-s001.zip › Supplementary File/Supplementary Table/Supplementary Table S2.pdf]

Supplementary Table S2. EMSA settings for different target genes

purM

1	2	3	4	5	6	7	8	9	Lane
0.0	17	0.0	0.0	0.0	0.0	0.0	0.0	0.0	Unlabeled target probe(pmol)
0.0	5.2	0.0	7.8	2.6	5.2	7.8	10	13	PurR protein(nmoL)
0.0	0.0	0.24	0.0	0.0	0.0	0.0	0.0	0.0	F1 antigen(μmoL)
0.0	0.0	0.0	1.4	0.0	0.0	0.0	0.0	0.0	Labeled 16S rRNA probe(pmol)
0.85	0.85	0.85	0	0.85	0.85	0.85	0.85	0.85	Labeled target probe (pmol)

purH

1	2	3	4	5	6	7	8	9	Lane
0.0	15	0.0	0.0	0.0	0.0	0.0	0.0	0.0	Unlabeled target probe(pmol)
0.0	42	0.0	63	21	42	63	84	105	PurR protein(nmoL)
0.0	0.0	0.24	0.0	0.0	0.0	0.0	0.0	0.0	F1 antigen(μmoL)
0.0	0.0	0.0	1.4	0.0	0.0	0.0	0.0	0.0	Labeled 16S rRNA probe(pmol)
1.2	1.2	1.2	0.0	1.2	1.2	1.2	1.2	1.2	Labeled target probe (pmol)

purE

1	2	3	4	5	6	7	8	9	Lane
0.0	11	0.0	0.0	0.0	0.0	0.0	0.0	0.0	Unlabeled target probe(pmol)
0.0	5.2	0.0	7.8	2.6	5.2	7.8	10	13	PurR protein(nmoL)
0.0	0.0	0.24	0.0	0.0	0.0	0.0	0.0	0.0	F1 antigen(μmoL)
0.0	0.0	0.0	1.4	0.0	0.0	0.0	0.0	0.0	Labeled 16S rRNA probe(pmol)
0.12	0.12	0.12	0.0	0.12	0.12	0.12	0.12	0.12	Labeled target probe (pmol)

guaB

1	2	3	4	5	6	7	8	9	Lane
0.0	21	0.0	0.0	0.0	0.0	0.0	0.0	0.0	Unlabeled target probe(pmol)
0.0	10	0.0	16	5.2	10	16	22	26	PurR protein(nmoL)
0.0	0.0	0.24	0.0	0.0	0.0	0.0	0.0	0.0	F1 antigen(μmoL)
0.0	0.0	0.0	1.4	0.0	0.0	0.0	0.0	0.0	Labeled 16S rRNA probe(pmol)
1.5	1.5	1.5	0.0	1.5	1.5	1.5	1.5	1.5	Labeled target probe (pmol)

carA

1	2	3	4	5	6	7	8	9	Lane
0.0	17.1	0.0	0.0	0.0	0.0	0.0	0.0	0.0	Unlabeled target probe(pmol)
0.0	10	0.0	16	5.2	10	16	22	26	PurR protein(nmoL)
0.0	0.0	0.24	0.0	0.0	0.0	0.0	0.0	0.0	F1 antigen(μmoL)
0.0	0.0	0.0	1.4	0.0	0.0	0.0	0.0	0.0	Labeled 16S rRNA probe(pmol)
0.96	0.96	0.96	0.0	0.96	0.96	0.96	0.96	0.96	Labeled target probe (pmol)

gcvT

1	2	3	4	5	6	7	8	9	Lane
0.0	22	0.0	0.0	0.0	0.0	0.0	0.0	0.0	Unlabeled target probe(pmol)
0.0	10	0.0	16	5.2	10	16	22	26	PurR protein(nmoL)
0.0	0.0	0.24	0.0	0.0	0.0	0.0	0.0	0.0	F1 antigen(μmoL)
0.0	0.0	0.0	1.4	0.0	0.0	0.0	0.0	0.0	Labeled 16S rRNA probe(pmol)
1.5	1.5	1.5	0.0	1.5	1.5	1.5	1.5	1.5	Labeled target probe (pmol)

katG

1	2	3	4	5	6	7	8	9	Lane
0.0	19	0.0	0.0	0.0	0.0	0.0	0.0	0.0	Unlabeled target probe(pmol)
0.0	5.2	0.0	7.8	2.6	5.2	7.8	10	13	PurR protein(nmoL)
0.0	0.0	0.24	0.0	0.0	0.0	0.0	0.0	0.0	F1 antigen(μmoL)
0.0	0.0	0.0	1.4	0.0	0.0	0.0	0.0	0.0	Labeled 16S rRNA probe(pmol)
0.75	0.75	0.75	0.0	0.75	0.75	0.75	0.75	0.75	Labeled target probe (pmol)

ssuE

1	2	3	4	5	6	7	8	9	Lane
0.0	8.8	0.0	0.0	0.0	0.0	0.0	0.0	0.0	Unlabeled target probe(pmol)
0.0	5.2	0.0	7.8	2.6	5.2	7.8	10	13	PurR protein(nmoL)
0.0	0.0	0.24	0.0	0.0	0.0	0.0	0.0	0.0	F1 antigen(μmoL)
0.0	0.0	0.0	1.4	0.0	0.0	0.0	0.0	0.0	Labeled 16S rRNA probe(pmol)
0.38	0.38	0.38	0.38	0.38	0.38	0.38	0.38	0.38	Labeled target probe (pmol)

purR

1	2	3	4	5	6	7	8	9	Lane
0.0	22	0.0	0.0	0.0	0.0	0.0	0.0	0.0	Unlabeled target probe(pmol)
0.0	5.2	0.0	7.8	2.6	5.2	7.8	10	13	PurR protein(nmoL)
0.0	0.0	0.24	0.0	0.0	0.0	0.0	0.0	0.0	F1 antigen(μmoL)
0.0	0.0	0.0	1.4	0.0	0.0	0.0	0.0	0.0	Labeled 16S rRNA probe(pmol)
1.5	1.5	1.5	0.0	1.5	1.5	1.5	1.5	1.5	Labeled target probe (pmol)

pyrD

1	2	3	4	5	6	7	8	9	Lane
0.0	9.4	0.0	0.0	0.0	0.0	0.0	0.0	0.0	Unlabeled target probe(pmol)
0.0	5.2	0.0	7.8	2.6	5.2	7.8	10	13	PurR protein(nmoL)
0.0	0.0	0.24	0.0	0.0	0.0	0.0	0.0	0.0	F1 antigen(μmoL)
0.0	0.0	0.0	1.4	0.0	0.0	0.0	0.0	0.0	Labeled 16S rRNA probe(pmol)
1.1	1.1	1.1	0.0	1.1	1.1	1.1	1.1	1.1	Labeled target probe (pmol)

serA

1	2	3	4	5	6	7	8	9	Lane
0.0	8.3	0.0	0.0	0.0	0.0	0.0	0.0	0.0	Unlabeled target probe(pmol)
0.0	5.2	0.0	7.8	2.6	5.2	7.8	10	13	PurR protein(nmoL)

0.0	0.0	0.24	0.0	0.0	0.0	0.0	0.0	0.0	F1 antigen(μmoL)
0.0	0.0	0.0	1.4	0.0	0.0	0.0	0.0	0.0	Labeled 16S rRNA probe(pmol)
1.5	1.5	1.5	0.0	1.5	1.5	1.5	1.5	1.5	Labeled target probe (pmol)

ogt

1	2	3	4	5	6	7	8	9	Lane
0.0	24	0.0	0.0	0.0	0.0	0.0	0.0	0.0	Unlabeled target probe(pmoL)
0.0	10	0.0	16	5.2	10	16	22	26	PurR protein(nmoL)
0.0	0.0	0.24	0.0	0.0	0.0	0.0	0.0	0.0	F1 antigen(μmoL)
0.0	0.0	0.0	1.4	0.0	0.0	0.0	0.0	0.0	Labeled 16S rRNA probe(pmoL)
1.5	1.5	1.5	0.0	1.5	1.5	1.5	1.5	1.5	Labeled target probe (pmoL

fur

1	2	3	4	5	6	7	8	9	Lane
0.0	7.1	0.0	0.0	0.0	0.0	0.0	0.0	0.0	Unlabeled target probe(pmoL)
0.0	13	0.0	20	6.6	13	20	26	33	PurR protein(nmoL)
0.0	0.0	0.24	0.0	0.0	0.0	0.0	0.0	0.0	F1 antigen(μmoL)
0.0	0.0	0.0	1.4	0.0	0.0	0.0	0.0	0.0	Labeled 16S rRNA probe(pmoL)
0.46	0.46	0.46	0	0.46	0.46	0.46	0.46	0.46	Labeled target probe (pmoL)

ybtA

1	2	3	4	5	6	7	8	9	Lane
0.0	16	0.0	0.0	0.0	0.0	0.0	0.0	0.0	Unlabeled target probe(pmoL)
0.0	13	0.0	20	6.6	13	20	26	33	PurR protein(nmoL)
0.0	0.0	0.24	0.0	0.0	0.0	0.0	0.0	0.0	F1 antigen(μmoL)
0.0	0.0	0.0	1.4	0.0	0.0	0.0	0.0	0.0	Labeled 16S rRNA probe(pmoL)
0.83	0.83	0.83	0.0	0.83	0.83	0.83	0.83	0.83	Labeled target probe (pmoL)

djlA

1	2	3	4	5	6	7	8	9	Lane
0.0	14	0.0	0.0	0.0	0.0	0.0	0.0	0.0	Unlabeled target probe(pmoL)
0.0	13	0.0	20	6.6	13	20	26	33	PurR protein(nmoL)
0.0	0.0	0.24	0.0	0.0	0.0	0.0	0.0	0.0	F1 antigen (μmoL)
0.0	0.0	0.0	1.4	0.0	0.0	0.0	0.0	0.0	Labeled 16S rRNA probe(pmoL)
0.76	0.76	0.76	0.0	0.76	0.76	0.76	0.76	0.76	Labeled target probe (pmoL)

The F1 antigen of *Y. pestis* served as a negative control protein.

A labeled segment of 16s rRNA gene served as a negative labeled probe.
